# Renal Transplant Experience in a Tertiary Care Center in Saudi Arabia: A Retrospective Cohort Study

**DOI:** 10.7759/cureus.23019

**Published:** 2022-03-10

**Authors:** Mohammed Tawhari, Moustafa S Alhamadh, Abdulrahman Alhabeeb, Mutlaq Almutlaq, Mansoor Radwi

**Affiliations:** 1 Nephrology, King Abdulaziz Medical City Riyadh, Riyadh, SAU; 2 Nephrology, King Abdullah International Medical Research Center, Riyadh, SAU; 3 Nephrology, King Saud Bin Abdulaziz University for Health Sciences College of Medicine, Riyadh, SAU; 4 Medicine and Surgery, King Saud Bin Abdulaziz University for Health Sciences College of Medicine, Riyadh, SAU; 5 Hematology, College of Medicine, University of Jeddah, Jeddah, SAU

**Keywords:** post transplant diabetes mellitus, kidney transplant recipient, solid organ transplant, living donor renal transplant, end stage kidney disease (eskd)

## Abstract

Background: Data on outcomes of renal transplantation in the Kingdom of Saudi Arabia (KSA) is limited. We describe renal transplant experience in one of the largest referral centers for renal transplants in KSA.

Methods:This is a retrospective cohort study of patients who underwent kidney transplantation at King Abdulaziz Medical City (KAMC) from January 2016 to July 31, 2019, with at least one year of follow-up post-transplantation.

Results: One hundred ninety-four individuals were identified and included in the analysis. The mean age of the renal transplant cohort was 45 years with an average pre-transplant body mass index of 26.1 kg/m^2^. The most common comorbidities were hypertension (77.3%) and diabetes mellitus (34.5%). The most common etiology of end-stage kidney disease (ESKD) was unknown (49.0%) followed by Lupus nephropathy (16.0%). Central venous catheters were the predominant dialysis access (56.2%). Living-related kidney donation was the commonest source of kidney transplantation (61.3%), followed by deceased donor renal transplantation (22.7%). Anti-thymocytes globulin (ATG) was the predominant induction agent (57.7%) and nearly all patients received tacrolimus-based maintenance immunosuppression. Mean serum creatinine at the time of discharge was 109 umol/l. Delayed graft function was observed in 6.7% of our patients. The most common medical complications were urinary tract infection (26.3%). Post-transplant surgical complications occurred at a low rate.

Conclusion:Our study demonstrated a successful renal transplant experience among a relatively healthy cohort and identifies potential gaps, particularly the high rate of central venous catheters, the frequent lack of clear etiology of ESKD, the low rate of pre-emptive transplant, and deceased donors. Further studies are needed to evaluate and close these gaps.

## Introduction

The prevalence of chronic kidney disease (CKD) has been increasing globally and is associated with impaired quality of life, morbidity, mortality, and a significant burden on the health care system [1,2]. This is largely attributed to the rising prevalence of diabetes mellitus (DM), hypertension (HTN), and obesity [2,3]. Renal transplantation is considered the ideal treatment strategy for patients with end-stage kidney disease (ESKD) [4,5]. Patients with renal transplantation have a higher survival rate compared to patients with other solid organ transplantation, mainly due to advancements in the field of renal transplantation and the availability of effective immunosuppressive agents [6]. Survival rate currently is reported to be 90%, 73.9%, 59.8%, 46.2%, and 36.7% in one, five, 10, 15, and 20 years, respectively [7,8]. However, patients who undergo renal transplantation may develop serious complications such as side effects of immunosuppression, renal artery or vein stenosis/thrombosis, pseudoaneurysms, urinary obstruction or leak, peri-transplantation fluid collections, acute tubular necrosis, graft rejection, psychosis, and malignancy [6,9,10]. Malignancies in renal transplant recipients are approximately threefold higher compared with the general population [11]. Infection is the leading culprit of mortality during the first year post renal transplantation, with urinary tract infection (UTI) being the most reported infection. In the first two months following renal transplantation, cytomegalovirus (CMV) is considered the predominant infection, accounting for approximately 46.5% of all infections [12].

A total of 11,509 renal transplantation surgeries has been carried out in the Kingdom of Saudi Arabia from 1979 to 2017 [13]. From a local perspective, limited data exist regarding outcomes of renal transplants in the Kingdom of Saudi Arabia. In this paper, we describe our center experience in kidney transplantation.

## Materials and methods

The study was conducted at King Abdulaziz Medical City (KAMC) in Riyadh, Saudi Arabia. KAMC is a tertiary hospital and is part of a government-funded multispecialty health system in Saudi Arabia. The data were collected after obtaining the IRB approval from King Abdullah International Medical Research Center (RC20/386/R).

We included patients who underwent kidney transplantation from January 2016 to July 31, 2019. All patients had to have at least one year of follow-up post-transplantation. Patients who had organs transplanted in addition to kidneys were also included. We excluded patients who were previously transplanted or underwent transplants outside of the Kingdom of Saudi Arabia.

We collected the following variables: age, gender, pre-transplant, and one-year post-transplant body mass index (BMI), smoking, cause of ESKD, type of dialysis, hospital length of stay, comorbidities prior to transplantation such as diabetes, HTN, coronary artery disease, cerebrovascular disease, complications post-transplant such as post-transplant diabetes, HTN, malignancy, and fracture, immunosuppressive regimen, and kidney function at the time of discharge after transplantation. Delayed graft function was also collected and was defined as the need for dialysis in the first week following transplantation.

Statistical Package for the Social Sciences (SPSS version 20; IBM, Armonk, NY, USA) was used for data analysis. Categorical variables were presented as frequencies and percentages whereas continuous variables were presented as mean ± standard deviation.

## Results

The demographics of our patients are shown in Table 1.

**Table 1:** Baseline characteristics MMF = Mycophenolate Mofetil, TAC = Tacrolimus, PRED = Prednisolone, CYCLO = Cyclosporine, ATG = Anti-Thymocyte Globulin

Variable	N	%
Age (years), (mean [SD])	45	(14.8)
Pre-transplant BMI (kg/m²), (mean [SD])	26.1	(5.1)
Gender (Male)	108	55.7%
Blood group		
A-	5	2.6%
A+	47	24.2%
B-	3	1.5%
B+	51	26.3%
AB-	1	0.5%
AB+	10	5.2%
O-	6	3.1%
O+	70	36.1%
Smoking	30	15.5%
Diabetes mellitus	67	34.5%
Diabetic neuropathy	24	12.4%
Hypertension	150	77.3%
Dyslipidemia	44	22.7%
Stroke	5	2.6%
Cancer	3	1.5%
Amputation	3	1.5%
Coronary artery disease	25	12.9%
Reduced ejection fraction (< 55%)	36	18.6%
Fracture prior to transplant	16	8.2%
Albumin (below 34 g/L)	25	1.0%
Renal Replacement modality		
Via catheter	109	56.2%
Via arteriovenous fistula/graft	55	28.4%
Pre-emptive	14	7.2%
Peritoneal dialysis	16	8.2%
Positive CMV status	97	50.0%
Positive EBV status	162	83.5%
Induction agent		
ATG	112	57.7%
Basiliximab	82	42.3%
Maintenance immunosuppression		
MMF/TAC/PRED	193	99.5%
MMF/CYCLO/PRED	1	0.5%

We identified 245 renal transplant recipients. We excluded 51 patients who underwent renal transplantation overseas, as they had a different immunological risk and better be described separately. Of those who were included in the analysis, 108 (55.7%) patients were males and 86 (44.3%) were females. The patients’ mean age was 45.01 ± 14.8 years with an average pre-transplant BMI of 26.13 ± 5.11 kg/m^2^ and a one-year post-transplant BMI of 28.67 ± 5.82 kg/m^2^. Only 30 (15.5%) patients were smokers. HTN was the most notable comorbidity (77.3%), followed by DM (34.5%), dyslipidemia (22.7%), and coronary artery disease (12.9%). Only a few patients had a history of stroke (2.6%), malignancy (1.5%), or amputation (1.5%). Causes of ESKD were unknown in almost half of the patients (49.0%). Lupus nephropathy (16.0%), DM (14.9%), and HTN (11.9%) were the most common identifiable causes of ESKD (see Table 2).

**Table 2:** Causes of end-stage kidney disease

Variable	N	%
Idiopathic/Unknown	95	49.0%
Systemic lupus erythematosus/Glomerulonephritis	31	16.0%
Diabetes mellitus	29	14.9%
Hypertension/Atrophied kidney	23	11.9%
Hereditary	7	3.6%
Other	9	4.6%

Most of the patients received dialysis through central venous catheters (CVCs, 56.2%), and arteriovenous fistula/graft (28.4%). Focusing on data specific to renal transplant, we found that living-related kidney donation was the commonest source of kidney transplantation (61.3%), followed by deceased donor renal transplantation (22.7%) and living-unrelated donation (16.9%). The overall rate of pre-emptive transplantation, defined as renal transplant prior to initiation of dialysis, was low (7.2%). Pre-transplant serology testing showed that 83.5% of our cohort was positive for Epstein-Barr virus (EBV) and 50% was positive for CMV. Of note, our center uses universal antiviral prophylaxis therapy that consists of three to six months of Valganciclovir post-transplantation.

Immunosuppressive induction therapy based on anti-thymocytes globulin (ATG) was the most prevalent (57.7%), followed by the Basiliximab-based regimen (42.3%). Most of our patients (99.5%) received maintenance immunosuppression that contained Tacrolimus along with prednisone and mycophenolate. It should be noted that our protocol uses a Basiliximab-based regimen for low immunological risk recipients and ATG for high immunological risk recipients. High immunological risk is defined as less than one haplotype match, previous kidney transplant, or high panel-reactive antibody (PRA) level. We do not use pre-transplant plasmapheresis, as patients with donor-specific antibodies (DSA) or blood group incompatibility is put through the kidney paired exchange program. Our maintenance regimen consists of low-dose prednisone, mycophenolate, and Tacrolimus, targeting a level of 7-10 ng/mL for the first three months after transplantation and 3-7 ng/mL for the subsequent months.

The post-transplant length of stay was 9.8±6.4 days, with 11 (5.7%) patients requiring intensive care unit admission following the renal transplant surgery. The average serum creatinine at the time of discharge following the renal transplant surgery was 109.1±97.5 µmmol/L (see Table 3).

**Table 3:** Characteristics related to transplant TAC = Tacrolimus, ATG = Anti-thymocyte globulin

Variable	N	%
Type of transplant		
Living-related donor	119	61.3
Living-unrelated donor	31	16
Deceased donor	44	22.7
Length of stay related to transplant (days), (mean [SD])	9.8	(6.4)
Intensive care stay related to transplant	11	5.7
Type of Immunosuppression		
Induction ATG (vs Baslixamab)	112	57.7
Maintenance TAC (vs. Cyclosporine)	193	99.5
Serum Creatinine at time of discharge post-transplant (µmmol/L), (mean [SD])	109.1	(97.5)

Complications related to the transplant are shown in Table 4.

**Table 4:** Post-transplant complications

Variable	N	%
Wound infection	2	1.0
Urine leak	3	1.5
Acute coronary syndrome	2	1.0
Urinary tract infection	51	26.3
Post-transplant diabetes	12	6.2
Post-transplant malignancy	2	1.0
Post-transplant fracture	7	3.6
Post-transplant psychiatric illness	7	3.6
Delayed graft function	13	6.7
Rejection of graft	14	7.2

UTI was diagnosed in 26.3% of our patients. Post-transplant surgical complications, including surgical site infection and lymphocele, were uncommon. Development of non-infectious complications post-transplant was rare with DM being the most common (6.2%), followed by fracture (3.6%), psychiatric illness (3.6%), and malignancy (1.0%). Delayed graft function was observed in 6.7% of our patients. Graft rejection in the first year occurred in 14 patients (7.4%). Of note, our transplant program does not perform surveillance allograft biopsies and all biopsies in our paper were done for clinical suspicion of rejection.

## Discussion

This study demonstrated a successful experience in renal transplantation at our center. Our cohort experienced a low rate of delayed graft function, rejection rate, and mortality. Surgical and medical complications were relatively lower than what has been reported in the literature [14-16] (e.g., wound infection and urine leak occurred at a rate of 1.0% and 1.5%, respectively), and acute coronary syndrome occurred at a rate of 1.0%. These observations were not surprising as our cohort were relatively healthy, young, with only a few comorbidities, and not morbidly obese at the time of transplantation.

While the transplant experience shows overall success in our center, we believe that a few important observations are needed to be addressed. First, we noticed that CVC, rather than arteriovenous fistula or graft, was the predominant hemodialysis access in these patients. The use of the CVC method can be explained by the availability of potential kidney donors at the time of initiation of dialysis that facilitated renal transplantation early, especially since the majority of our renal transplant recipients had living-related donors. 

Second, only a few patients received pre-emptive transplantation, despite the availability of a living related donor for over half of this transplanted cohort. Pre-emptive renal transplantation has been shown to improve patient and graft survival [17]. We also observed that the rate of deceased donor transplantation was only 22.7%. These observations signify the need to increase awareness about early referrals for pre-emptive renal transplantation particularly when donors are available, and to encourage organ deceased donations. Thus, we advocate for establishing an information pathway directed to physicians, patients, and families, organizing national campaigns, and establishing outreach nephrology transplant programs.

Third, the cause of ESKD was not identified in almost half of our patients. However, it should be noted that many of the transplanted patients in our cohort were referred from other healthcare institutes. It is possible that these patients were arriving there at a late stage, where identifying the cause of ESKD is less likely to alter the therapy, or simply not possible. It is also possible that these institutes did not have the ability to conduct comprehensive investigations to identify the cause of ESKD or had limited capability to keep medical records. Further studies are needed to better address these issues.

Fourth, only 14.9% of our cohort were labeled to have diabetic nephropathy, and only 34.5% of those who underwent renal transplantation were diabetic. Diabetic nephropathy is the leading cause of ESKD worldwide, and diabetes is one of the most common chronic diseases in Saudi Arabia [18]. However, we believe that this paradox may be because patients with diabetic nephropathy are either under-referred or have been excluded due to comorbidities. This calls for further studies to examine this finding.

Fifth, the rate of delayed graft function, defined as dialysis requirement within the first post-transplantation week, was relatively low at 6.7%, which is less than previous studies [19]. Most of the renal transplants in this cohort were through living donation, in which delayed graft function is rare as it usually takes place in an ideal planned manner and avoids any prolonged cold ischemia time during transplantation.

Sixth, it is reassuring that the rate of acute rejection in our cohort is similar to the international reports. This is expected since most of this cohort received living-related renal transplants, induced with ATG and maintained on Tacrolimus and antimetabolites [20].

Seventh, the most common long-term complication in our cohort was post-transplantation diabetes (6.2%), similar to what has been reported in the literature [21]. The second most common complications were post-transplant psychiatric illness and post-transplant fracture, occurring at a rate of 3.6% each. Interestingly, post-transplantation diabetes, post-transplant psychiatric illness, and post-transplant fracture are all associated with corticosteroids dosing. Our center steroids protocol consists of 500 mg of intravenous methyl-prednisone given prior to the surgery followed by 250 mg of intravenous methyl-prednisone in each of the following two days, then the patient goes home on 25 mg of oral prednisone tapering down by 5 mg every week until reaching 5 mg. Most patients will continue 5 mg of oral prednisone throughout the post-transplantation course. We feel that we can mitigate these complications by using a lower steroids dosage or by tapering steroids rapidly, particularly in high-risk populations (e.g., obese patients or those with prediabetes or impaired glucose tolerance). We are planning to examine this in the context of a randomized clinical trial within two years and are currently in process of designing a protocol for it. 

Our study has strengths and limitations. The strengths of this study are: 1) it provides a comprehensive descriptive analysis from a referral center for renal transplant the Kingdom of Saudi Arabia, 2) we have identified gaps and areas for further research and improvement, particularly early referral for pre-emptive transplantation, improving the deceased donor pool, transplantations of diabetic people, the need for special emphasis in identifying and treating the primary cause of ESKD, and 3) our study also calls for the better steroid-using strategies among renal transplant population. Limitations include it is an observational study, conducted in a single center, has relatively a small sample size, and did not include the serum creatinine level past the time of discharge.

## Conclusions

This study demonstrated a successful renal transplant experience among a relatively healthy cohort and identifies potential gaps, particularly the high rate of CVCs, the frequent lack of clear etiology of ESKD, the low rate of pre-emptive transplant and deceased donors. Prospective studies are needed to evaluate and close these gaps. The study also showed that our outcomes are comparable to the international reports.

## References

[1] Ammirati AL (2020). Chronic kidney disease. Rev Assoc Med Bras (1992).

[2] Lv JC, Zhang LX (2019). Prevalence and disease burden of chronic kidney disease. Adv Exp Med Biol.

[3] Matsushita K, van der Velde M, Astor BC (2010). Association of estimated glomerular filtration rate and albuminuria with all-cause and cardiovascular mortality in general population cohorts: a collaborative meta-analysis. Lancet.

[4] Hogan J, Pietrement C, Sellier-Leclerc AL (2017). Infection-related hospitalizations after kidney transplantation in children: incidence, risk factors, and cost. Pediatr Nephrol.

[5] Lai X, Chen G, Qiu J, Wang C, Chen L (2014). Recipient-related risk factors for graft failure and death in elderly kidney transplant recipients. PLoS One.

[6] Mohammadi MH, Salarzaei M, Parooie F (2019). Neurological complications after renal transplantation: a systematic review and meta-analysis. Ther Apher Dial.

[7] Galindo Sacristán P, Pérez Marfil A, Osorio Moratalla JM (2013). Predictive factors of infection in the first year after kidney transplantation. Transplant Proc.

[8] Banas B, Krämer BK, Krüger B, Kamar N, Undre N (2020). Long-term kidney transplant outcomes: role of prolonged-release tacrolimus. Transplant Proc.

[9] Inci MF, Ozkan F, See TC, Tatli S (2014). Renal transplant complications: diagnostic and therapeutic role of radiology. Can Assoc Radiol J.

[10] Abbott KC, Agodoa LY, O'Malley PG (2003). Hospitalized psychoses after renal transplantation in the United States: incidence, risk factors, and prognosis. J Am Soc Nephrol.

[11] Sprangers B, Nair V, Launay-Vacher V, Riella LV, Jhaveri KD (2018). Risk factors associated with post-kidney transplant malignancies: an article from the cancer-kidney international network. Clin Kidney J.

[12] Traynor C, Jenkinson A, Williams Y (2012). Twenty-year survivors of kidney transplantation. Am J Transplant.

[13] (2022). Introduction and overview of Saudi Center For Organ Transplantation (SCOT). https://www.scot.gov.sa/testtube/News/News/15.

[14] Harris AD, Fleming B, Bromberg JS (2015). Surgical site infection after renal transplantation. Infect Control Hosp Epidemiol.

[15] Buresley S, Samhan M, Moniri S, Codaj J, Al-Mousawi M (2008). Postrenal transplantation urologic complications. Transplant Proc.

[16] Felix R, Saparia T, Hirose R (2016). Cardiac events after kidney transplantation according to pretransplantation coronary artery disease and coronary revascularization status. Transplant Proc.

[17] Kasiske BL, Snyder JJ, Matas AJ, Ellison MD, Gill JS, Kausz AT (2002). Preemptive kidney transplantation: the advantage and the advantaged. J Am Soc Nephrol.

[18] Narres M, Claessen H, Droste S, Kvitkina T, Koch M, Kuss O, Icks A (2016). The incidence of end-stage renal disease in the diabetic (compared to the non-diabetic) Population: a systematic review. PLoS One.

[19] Quiroga I, McShane P, Koo DD, Gray D, Friend PJ, Fuggle S, Darby C (2006). Major effects of delayed graft function and cold ischaemia time on renal allograft survival. Nephrol Dial Transplant.

[20] Hart A, Smith JM, Skeans MA (2020). OPTN/SRTR 2018 Annual Data Report: Kidney. Am J Transplant.

[21] Kasiske BL, Snyder JJ, Gilbertson D, Matas AJ (2003). Diabetes mellitus after kidney transplantation in the United States. Am J Transplant.

